# Detecting the Seeds of *Nassella neesiana* in Large Round Hay Bales, by Means of Non-Destructive Core Sampling

**DOI:** 10.1371/journal.pone.0137343

**Published:** 2015-09-08

**Authors:** Sandra Weller, Singarayer Florentine, James Sillitoe, Charles Grech, David McLaren, Bhagirath Singh Chauhan

**Affiliations:** 1 Centre for Environmental Management, Faculty of Science and Technology, Federation University Australia, Mount Helen, Victoria, Australia; 2 Research Services, Federation University, Mount Helen, Victoria, Australia; 3 Department of Environment and Primary Industries, Bundoora, Victoria, Australia; 4 Queensland Alliance for Agriculture and Food Innovation (QAAFI), The University of Queensland, Toowoomba, Queensland, Australia; Huazhong University of Science and Technology, CHINA

## Abstract

In the last three decades or so there has been a significant increase in fodder trading, both in terms of the quantity of fodder traded and in terms of its economic value to the industry. Often, this fodder type may be supplied free of charge to graziers in distress due to circumstances that follow natural disasters such as bushfires, drought, and flood. However, because of the obvious urgency arising from these situations, it is suspected that much relief fodder may unintentionally pose an elevated risk for dispersal of weeds since it may be supplied from pasture not normally used for trade in fodder, and therefore is of unknown quality. Previous destructive method to detect weed propagules in bales of fodder are cumbersome, time consuming and of limited ecological and statistical value. Therefore, objective of this paper was to development of a convenient method to assess round pasture hay bales for the presence of weed propagules, to prevent unintentional spread of noxious species in hay bales. To examine this objective known quantity of seeds were added in a series of distributions to bales of seed free pasture hay, and a positive correlation for the amount of seed added per bale with that recovered in core samples was observed. Whilst the number of seeds detected per bale varied according to the distribution of seeds within the bales and the number of cores analysed, the absolute detection of seeds suggests that this sampling method is worthy of further examination. In addition, a pragmatic estimation of bale remnants after stock feeding has been investigated to more closely estimate the potential size of the remaining seed bank. The authors propose that development of this approach is timely, in the light of future climatic uncertainty driving extreme weather events that increase the need for relief fodder, which can be a potential vector for the spread of noxious weed seeds.

## Introduction

Agricultural weeds pose significant problems for primary producers. Effects of weed infestation range from reduced yields due to competition for nutrients and water with cash crops, to the adulteration of the quality of harvested crop material, suggesting that the control of weed dispersal is absolutely essential. This investigation relates to one of the many pathways that have been recognised for the inadvertent mechanisms that contribute to weed dispersal [1, 2]. Whilst it is accepted that provisioning fodder for livestock is vital for many agricultural enterprises in Australia, during the last 30 years or so there has been a significant increase in fodder trading, both in terms of the quantity of fodder traded and its economic value to the industry [3]. Of particular interest here is that the broadest definition of ‘traded fodder’ includes that of relief fodder, which is needed at short notice during emergency situations [4]. Often, this fodder type may be supplied free of charge to graziers in distress due to circumstances that follow natural disasters such as bushfires, drought, and flood [5, 6]. However, because of the obvious urgency arising from these situations, it is suspected that much relief fodder may unintentionally pose an elevated risk for dispersal of weeds since it may be supplied from pasture not normally used for trade in fodder, and therefore is of unknown quality. Such uncontrolled areas can be cut, baled and, within a short time frame, transported over large distances before control inspections can be reasonably instituted [7]. In addition, burned and distressed areas of ground in the regions receiving the bales can be ideal locations for establishment of new weed infestations, which increasingly exacerbates the problem [6].

The world-wide increase in trade in fodder within the industry has already prompted measures to mitigate the possibility of unintentional weed dispersal. In the United States of America, for example, weed-free fodder programs have been introduced, where intending suppliers of fodder must apply for certification. They are required to ensure that no noxious weed seeds are included in traded fodder bales, which commonly is assured by harvesting only weed-free pasture [8–10]. However, in Australia, where the trade in fodder is of a considerable scale, weed-free fodder programs have only recently been initiated and the approaches for assurance of weed-free status of fodder vary from state to state. For example, in New South Wales, suppliers of fodder are required to give written assurances of the noxious weed-free status of bales [11]. In South Australia, on the other hand, there is no requirement for assurances of weed-free status to be given in writing [12]. At the present time, however, there appears to be no certified rapid assessment method to test these assurances.

It is against this already uncertain quality assurance background that the problem of emergency fodder, a historical feature of Australian agriculture, becomes particularly important. Exacerbating this problem is the possibility that, with the increasing likelihood of climate change-driven weather events, more extreme conditions of fire and flood will magnify this need for emergency fodder. Given that the quality control for normal trading cycles are only just under development, the likelihood of increasing frequency and extent of emergency events will pose even more serious questions. How can material taken from uncontrolled pastures be screened effectively to prevent weed dispersal when time frames are short and assay procedures are underdeveloped?

It appears that studies that specifically aim to determine which and how many weed seeds are present in hay bales are few. Despite much anecdotal evidence of weed seed dispersal within hay bales, this area of knowledge appears to be only at an early stage of development in rigorous scientific terms. Two studies that specifically investigated the presence of weed seeds in baled material were found in the literature [5, 13]. Each used destructive sampling methods of single bales, or a significant proportion of a single bale, to identify and enumerate seeds. In these studies, the entire bale, or a sampled portion comprising at least 25% of the bale, was dismantled completely and sieved to recover seed-bearing material. However, it is estimated that applying this approach to bales of a large size, for example those greater than 200 kg, would be likely to need several hours to obtain samples for analysis. Additionally, in these reports, germination was relied upon to identify at least some of the weed species that were present. This process typically requires a minimum of two to three weeks to obtain seed identification, and would, of course, not identify them if their seeds do not germinate due to dormancy or other reasons. While each of these studies demonstrated conclusively that weeds of noxious species are definitely dispersed in hay bales, the methods used for identification are unsuitable for rapid, economic, and efficient sampling and analysis.

Another issue that can be identified from these studies is the use of single bales for analysis. A single bale represents, at best, a relatively small and discrete area of a pasture, being taken from perhaps only a few tens of square metres. If this area of pasture by chance does not contain a particular noxious weed, then it would be undetected in the harvested lot. This chance element clearly poses a significant risk of unintentional weed dispersal in hay bales from an extended uncontrolled pasture. We maintain that it is preferable that an assumption of heterogeneous distribution of weed seed be made in such emergency pastures, and an approach to analysis be made to increase the likelihood of detection of concentrated spots of weed presence in bales from a particular lot.

It is agreed that, in order to obtain a representative mean value of some parameter of interest, a large number of small-sized samples are more likely to give a more reliable estimate rather than using a small number of large-sized samples [14]. Similarly, the assessment of the composition of a large area of a pasture by obtaining multiple, small-sized samples will increase the probability of detection of a weed infestation [15] in contrast to sampling a small area intensively. In the context of this investigation, the development of a method to take multiple small samples from multiple bales constituted from a pasture would be more desirable method to obtain a representative estimate of the composition of a pasture than with a single hay bale tested to destruction. We suggest that such an approach would significantly increase the probability of detection of ‘patchy’ weed seed infestations, facilitating the prevention of noxious weed dispersal.

We have noted that researchers have investigated the problem of obtaining representative samples from baled commodities, including estimates of wool parameters for shrinkage analysis [16, 17] and analysing fodder for levels of feed quality [18–21]. At least one group of researchers have specifically commented on the efficiency and reliability with which core samples could be obtained from baled fodder, with the use of suitable sampling equipment [18]. Some other related issues of relevance to the current research were also identified. Specifically, the question of ‘what is an appropriate number of cores per bale for analysis?’ has been the subject of a number of investigations [22, 23]. Whilst most researchers in this area recommend that one or two samples per bale is sufficient [24, 25], previous work has investigated homogeneous bales composed of a single species, usually alfalfa (*Medicago sativa* L.).

In bales composed of a mixture of species, such as the pasture hay bales of interest to this investigation, a lack of homogeneity of composition is more likely to occur due to the ‘patchy’ mixture of fodder species and weeds which may be present. In these heterogeneous bales, it is highly unlikely that only two cores per bale would be sufficient for reliably detecting weed seed presence in hay bales, especially with large round bales. We therefore were concerned to estimate the minimum number of cores for sampling a large round bale which would give some level of confidence about the presence or absence of weed seeds in the bale. A method of core sampling round pasture hay bales, with the objective of determining the appropriate number of cores to obtain a reliable estimate of weed seed presence or absence, is described in an experiment that was carried out in 2012 in Victoria, Australia.

To undertake this investigation into testing large round bales, we selected a species whose seeds are easily identifiable and available in large numbers. This selection allowed ease of detection of the seeds by visual detection and calculation of probabilities of detection by various methods of core sampling. *N*. *neesiana* is a noxious grass weed that is suitable for this type of investigation, since its seeds are easily collected by hand from the flowering stems just prior to full ripeness, and the seeds have a distinctive morphology that is easily identified when found in core samples of hay bales.

It is also strongly suspected that this weed has been dispersed in hay bales produced from properties in the northern outer Melbourne region of Victoria, Australia (C. Grech, pers. com.), and this added a strong pragmatic element to the choice. This species produces hard, unpalatable flowering stems during the spring and early summer with sharp, needle-like seeds that cause significant animal welfare issues [26]. It has a versatile reproductive strategy producing more than one type of seed [27]. During the so-called ‘masting’ years [28], each plant produces hundreds of panicle seeds periodically, increasing the probability of its dispersal. In Victoria, record breaking rainfalls occurred during 2010 following more than 10 years of lower than average rainfall [29], and as a result, infestations of *N*. *neesiana* occurring in the Melbourne region seeded prolifically, adding to the urgency of this sort of investigation.

The detection of the potential numbers of weed seeds which contaminate bales of relief fodder is only the beginning of the solution to this problem. From this issue arises a deeper consideration of the risk of seed dispersal where some fraction of these bales is not consumed by livestock, but remains on the ground in the paddock where they are fed. Two further questions that may be posed are:

How much seed is likely to remain in the pasture, after livestock have been fed weed-seed contaminated bales? What is the likelihood of this seed starting a new infestation?

To answer the first question, data for the proportion of feed waste due to the consumption of large round bales by livestock was obtained from the literature. This enabled calculations to be made for the numbers of seeds which are likely to remain in pastures, following the consumption of weed-seed contaminated bales. This information is used, together with information obtained from the study of weed-seed soil seed banks, to establish the likelihood of new infestations of *N*. *neesiana* arising from the use of contaminated relief fodder. While information from the literature is somewhat limited for both of these questions, the information that is available can give a starting point to evaluate the risk of dispersal of this weed through relief fodder.

Belyea *et al*. (1985) and Buskirk *et al*. (2003) conducted experiments to determine the quantity of wastage from feeding large round bales to cattle. The former authors investigated the effect of storage of feed prior to feeding on wastage. The latter investigated the effect of devices used to hold bales, while livestock fed on the bales contained therein, on fodder wastage and feeding behaviour. The amount of fodder wasted varied from 4% to 25% [30, 31].

## Materials and Methods

(i) “Souters” property in Somerton Road, Greenvale, Victoria—This is a private property. We obtained permission from the landholder to collect *Nassella neesiana* seeds and conduct study. (ii) Dr. David McLaren (Department of Primary Industry’s) provided a permission to store and hay bales and bales core sample in the Department of Primary Industry, Atwood. (iii) We can confirm that the field studies did not involve endangered or protected species. *Nassella neesiana* is a weed species, we have permit to conduct research on this species. The permit was grant by the Department of Primary Industry.

### Detecting weed seeds in round bales

*Nassella neesiana* seeds were harvested by hand from mature seed-heads during November 2010 from the “Souters” property in Somerton Road, Greenvale, Victoria. Seeds from more than 300 individual plants were bulked to provide a representative seed sample reservoir. The cleaned seeds were stored in labelled air tight container at room temperature at Federation University, Ballarat, Victoria, Australia until early 2012. In March 2012, just prior to commencement of field work, weighed lots of seed were placed into paper bags and heat-killed in a laboratory oven set at 105°C for three days. It had been determined previously that this temperature and time of exposure could reduce the viability of the seeds of this weed to zero (S. Weller, unpublished data). These heat-killed seeds were used to eliminate the possibility of dispersal of viable seed within the baling machine that was used for this experiment and to eliminate risk of viable seed being left behind at the site where fieldwork was conducted. This paddock was not known to contain any existing infestation of *N*. *neesiana* and it was intended that the bales would be unrolled and left at this site when core sampling of bales was completed.

The field work, during which seeds were added to the bales and the bales core sampled, was conducted between April and June 2012 at the Department of Primary Industry’s research facility at Attwood, Victoria, Australia. The bales of pasture hay that were used for this investigation were sourced from a property (37°35’05.93”S, 144°50’12.05”E) that was known to not have any *N*. *neesiana* infestation. The bales consisted of mixed pasture species dominated by exotic grasses, mainly *Dactylus glomerata* L. (Cocksfoot) and *Phalaris aquatica* L. (Toowoomba canary grass). The bales were rolled out into windrows prior to the addition of seeds, and varying weights of *N*. *neesiana* seed, (50, 150, 250 and 1000 g) were placed along the windrows in three different distribution patterns; (i) concentrated towards the beginning of the windrow, (ii) evenly dispersed along the windrow and (iii) concentrated towards the end of the windrow (Table 1). Consequently, after the windrows were re-rolled, the seeds were concentrated in the bales to varying depths (i) towards the centre of the bale, (ii) evenly throughout the bale and (iii) towards the outer section of the bale.

**Table 1:** Experimental design of seeds added to bales.

Amount of seed (g) per bale	Dispersal of seeds in bale	Number of bales	Number of cores per bale	Total number of cores
50	3 distribution patterns: Inner, Even, Outer	3	15	45
100		3	15	45
150		3	15	45
250		3	15	45
1000		2	15	30
Control	n/a	3	15	45

### Core sampling bales

**Fig 1:**
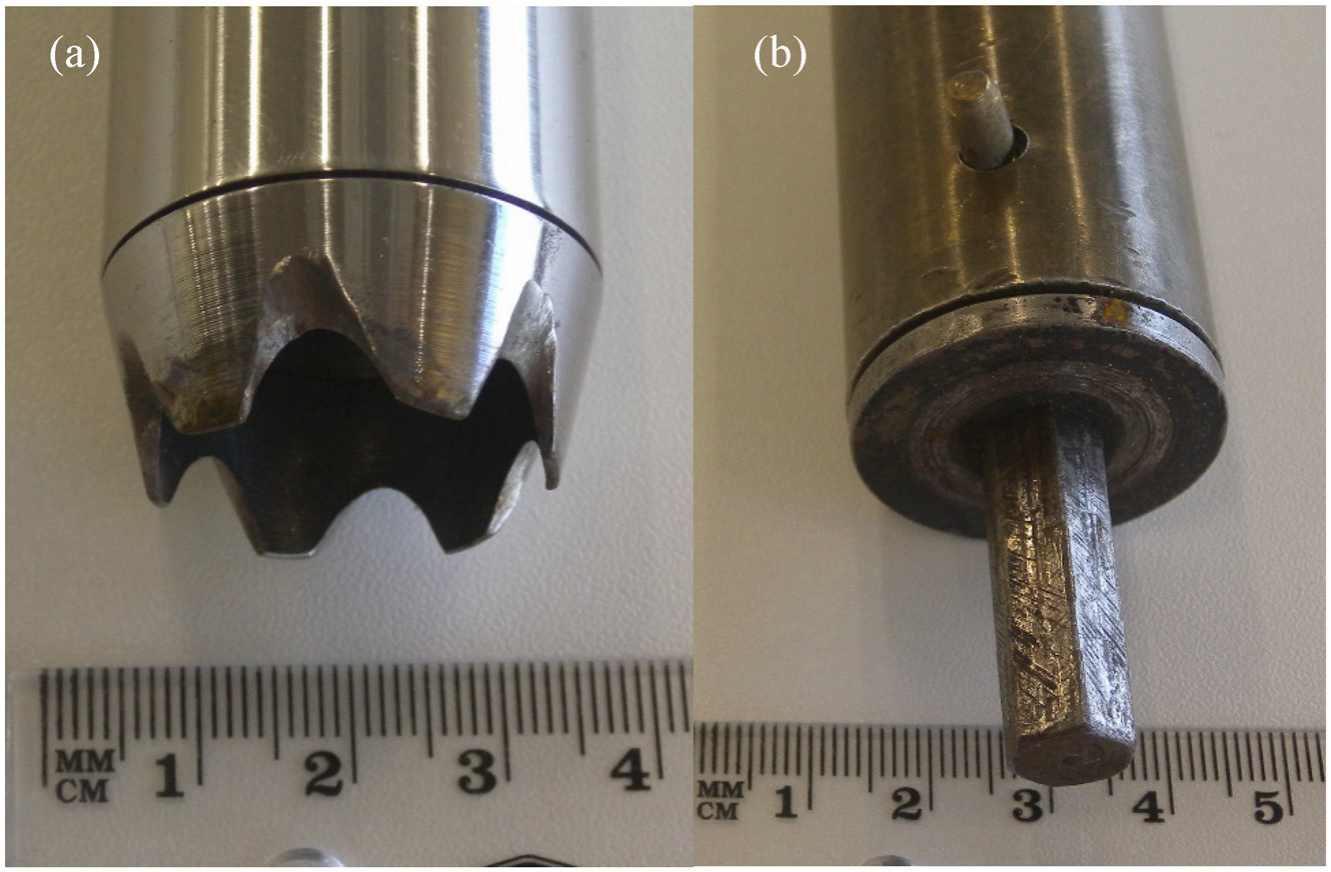
(a) and (b) Corer used for obtaining core samples from bales. (a) Cutter end of modified hay corer, and (b) drill chuck end of modified hay corer.

After being re-rolled, the bales were core sampled using a stainless steel hay corer (Fig 1(a) and (b)) [32] driven by an electric drill. Bales were systematically cored across the rolling surface in a straight line and to the centre of each bale. A total of 15 cores were taken from each bale, spaced approximately 3 to 5 cm apart. In this manner, the entire width of each bale was sampled, as was the entire length of the windrow. Each core sample was placed in a labelled paper bag and transported to the Federation University for further analysis. To prevent samples from becoming mouldy, they were dried in an oven for one week at 40°C and stored in the laboratory at room temperature until analysed.

The hay corer used was modified since the original configuration of the corer tube was of insufficient length to enable the corer to reach the centre of the round hay bales. The corer was to lengthened to 110 cm by removing the cutter and chuck ends (Fig 1(a) and (b)) and attaching each to either end of a longer steel tube of the same gauge (width and wall thickness) as the original.

Two issues pertaining to the coring equipment were encountered during the course of the field work. The first of these was that the cordless drill, which had been purchased for this study, was found to be useful only for taking relatively few samples per sampling session. Despite purchase of a powerful drill that was available at a reasonable price (AU$550, Milwaukee® brand 18V cordless, Model no. HD 10 PD, with a 3 amp/hr battery), it was able to be used for a maximum of two to three bales (30 to 45 cores) before the battery became too flat to continue to turn the corer. Denser bales made this likely to occur more rapidly. An extra battery had been purchased, however these needed to be recharged for a minimum of one to two hours each. It was subsequently decided to purchase a corded drill (Hitachi® Model D 13VG, 710W), a generator, and an extension cord to obtain the core samples (630 cores in total). This allowed for at least six to eight bales to be cored in a single session before the operator became too fatigued.

### Examination of cores

All core samples were examined, including the control those from bales to which no seed was added. Each sample was initially sieved, with a 1 mm mesh sieve, into a plastic tray, approximately 30 x 40 cm and 15 cm deep, to determine whether any *N*. *neesiana* seeds were present. This mesh size had been previously found to retain seeds of this weed and hold them securely, separating them from the bulk of cored material. Material that did not pass through the sieve was placed into a second tray for visual examination, and the fine material that had passed through the mesh was also examined for seeds of *N*. *neesiana* that had not filled out and which were able to pass through the sieve mesh. These could be located among this fine material upon subsequent examination, and a small numbers of seeds were found in this manner. By systematically separating out approximately 2 to 3 cm^3^ volumes of cored material and spreading this out to separate the individual particles, seeds could be distinguished from stem, leaf and seeds of non-target species. Since the seeds of *N*. *neesiana* are elongated and have a sharp tip, some of these were found to have pierced and lodged in fragments of stems and leaves that made up the bulk of the hay bales. These embedded seeds could not be detected by capture within the mesh of the sieve, which made it necessary to manually examine the sieved material to obtain a full count of seeds in the core samples.

### Statistical analysis

To find whether there was a cut-off concentration of seed below which it was not possible to detect seeds in these bales, the foregoing results were analysed by a simple regression analysis in SPSS.

## Results and Discussion

### Detection and quantification of seeds in sampling cores

**Fig 2:**
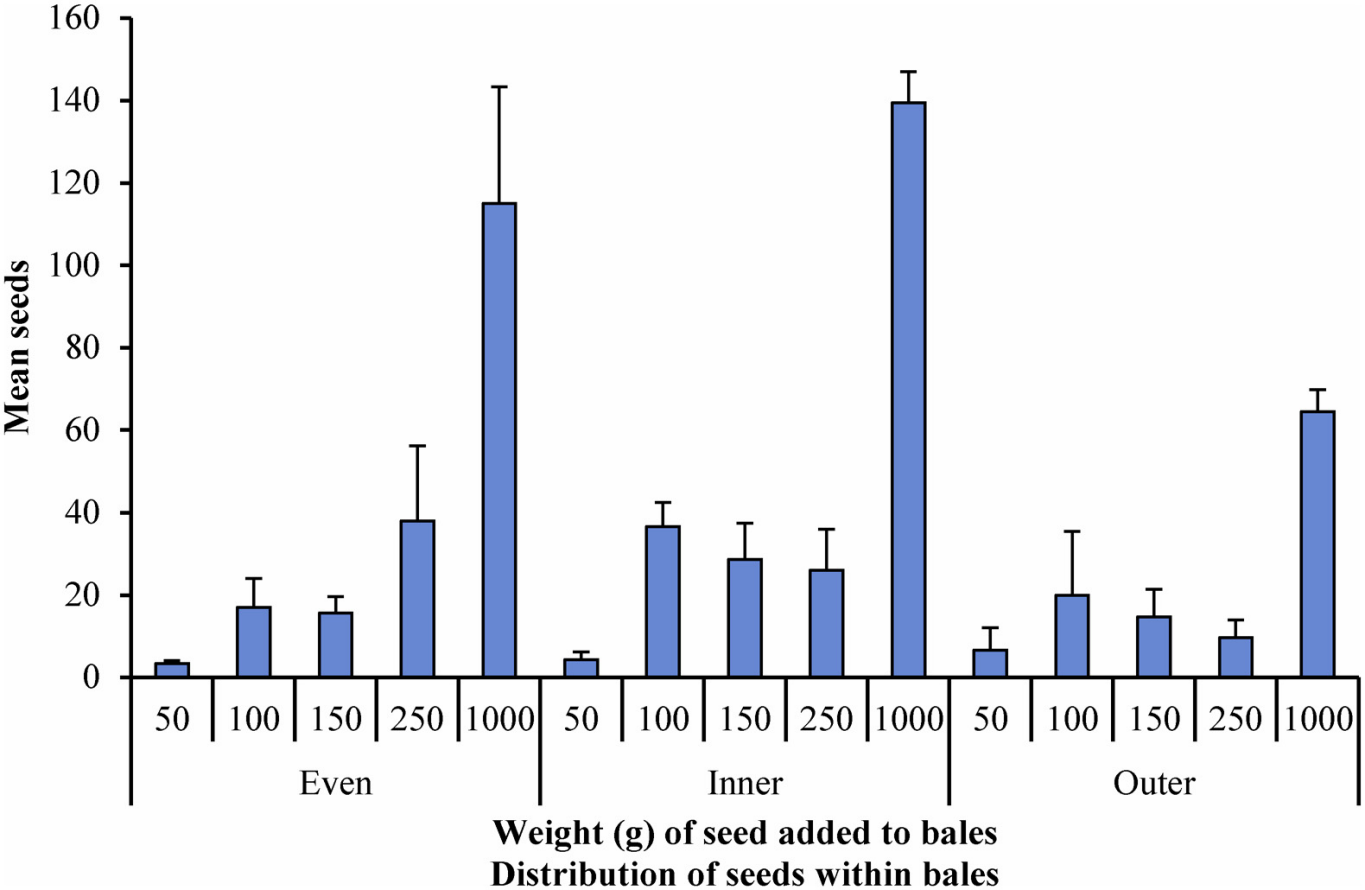
Results of bale analysis, based on 15 cores per bale.

There are two aspects of sampling of particular interest to this investigation. First, and most importantly, there is the question of absolute detection of contaminating seeds within the bale. Second, we are interested in whether this sampling regime can be used to predict the seed density within a bale with any certainty. As seen in Fig 2, for each mode of seed distribution and level of seed addition, there was qualitative detection of *N*. *neesiana* seed. However, the correlation of the number of seeds found within the sample with the total weight of seeds added to the bales was understandably poor in each case (S1 Dataset).

The fit of each regression (*R* ^*2*^) indicates a better than 0.50 fit of the data for all distributions, indicating that more than half of the variation in the results can be accounted for by the regression results and less than half is due to random error(S2 Dataset). Inner distribution gave the highest *R* ^*2*^ value and Outer distribution the lowest (Table 2).

**Table 2:** Results of regression for numbers of seeds, 15 cores per bale.

Regression Statistics	Even	Inner	Outer
*R* ^*2*^	0.71	0.86	0.54
Adjusted *R* ^*2*^	0.69	0.84	0.50
Observations	14	14	14
Intercept (*b*_*0*_)	2.48 ± 4.24	6.31 ± 3.12	5.30 ± 3.06
Intercept (*b*_*1*_)	0.11± 0.01	0.13± 0.01	0.06± 0.01
Regression P-value	0.000	0.000	0.003
*b*_*0*_ Lower 95%	-16.00	-7.3	-8.01
*b*_*0*_ Upper 95%	20.96	19.91	18.61
*b*_*1*_ Lower 95%	0.07	0.10	0.02
*b*_*1*_ Upper 95%	0.16	0.16	0.09

The seed detection cut-off figures, indicated by the intercept (b_0_), were 2.48 ± 4.24 seeds for Even distribution, 6.31 ± 3.12 seeds for Inner distribution, and 5.30 ± 3.06 seeds for Outer distribution. This indicates that it is possible to detect a minimum number of seeds in these bales of between approximately 2 and 6 seeds (rounded up).

### Detection of seeds in fewer than 15 cores per bale

The equipment that was used to obtain the core samples allowed for at least 15 cores per bale to be collected in less than one hour, generally as quickly as 20 to 30 minutes. Therefore, three bales can be sampled in less than an hour and a half. However, if a larger number of bales were available to be sampled, this intensive sampling regimen would be too time consuming. In the circumstance where there are more than 20 bales in a lot, for example, it would be more practical to sample a larger number of bales less intensively. This would effectively increase the area of the pasture sampled for the presence of weeds and therefore increase the likelihood of detecting weed seeds in the bales.

To determine the effect of reducing sampling effort per bale, the data obtained for 15 cores per bale was interrogated for 7 and 5 cores, respectively. Table 3 shows how data from core samples for 15 cores per bale were systematically selected to represent even spacing of cores across the entire bale, for approximately half and one third of the cores. By commencing with the centremost core (core number 8) and choosing every second core from this point outward to the edge of the bale for the 7 cores per bale sub-sample, the even numbered cores were analysed. For the 5 core sub-sample, core number 8 and every third core either side, core numbers 2, 5, 11, and 14, were analysed.

**Table 3:** Individual cores selected for statistical analysis by number for total cores (15), approximately one half of the total and one third of the total.

Core number	1	2	3	4	5	6	7	8	9	10	11	12	13	14	15
15 cores	x	x	x	x	x	x	x	x	x	x	x	x	x	x	x
7 cores		x		x		x		x		x		x		x	
5 cores		x			x			x			x			x	

**Fig 3:**
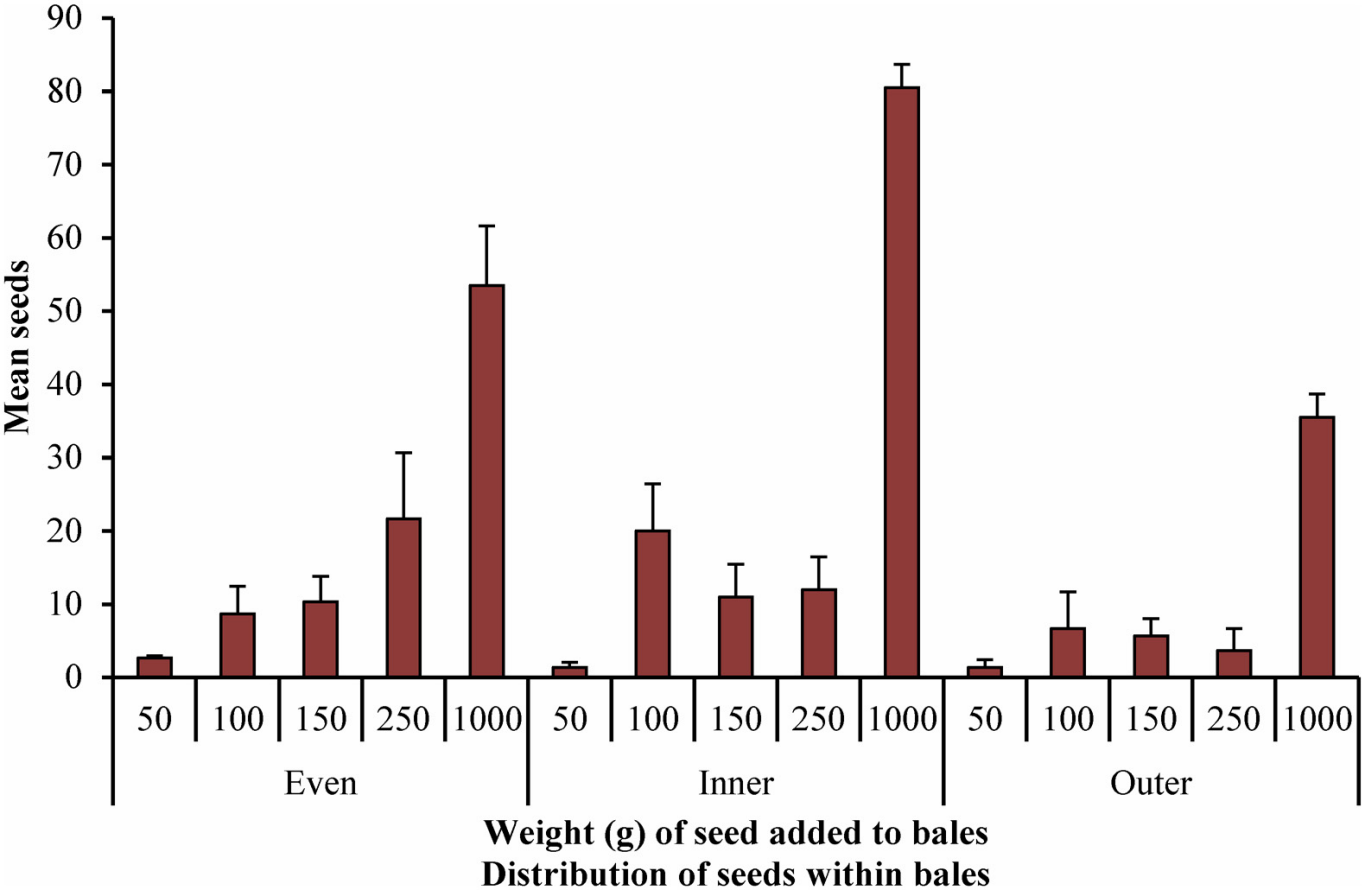
Numbers of seeds found per bale in bales to which varying weights of seed were added, in three different distribution patterns, 7 cores per bale.

**Fig 4:**
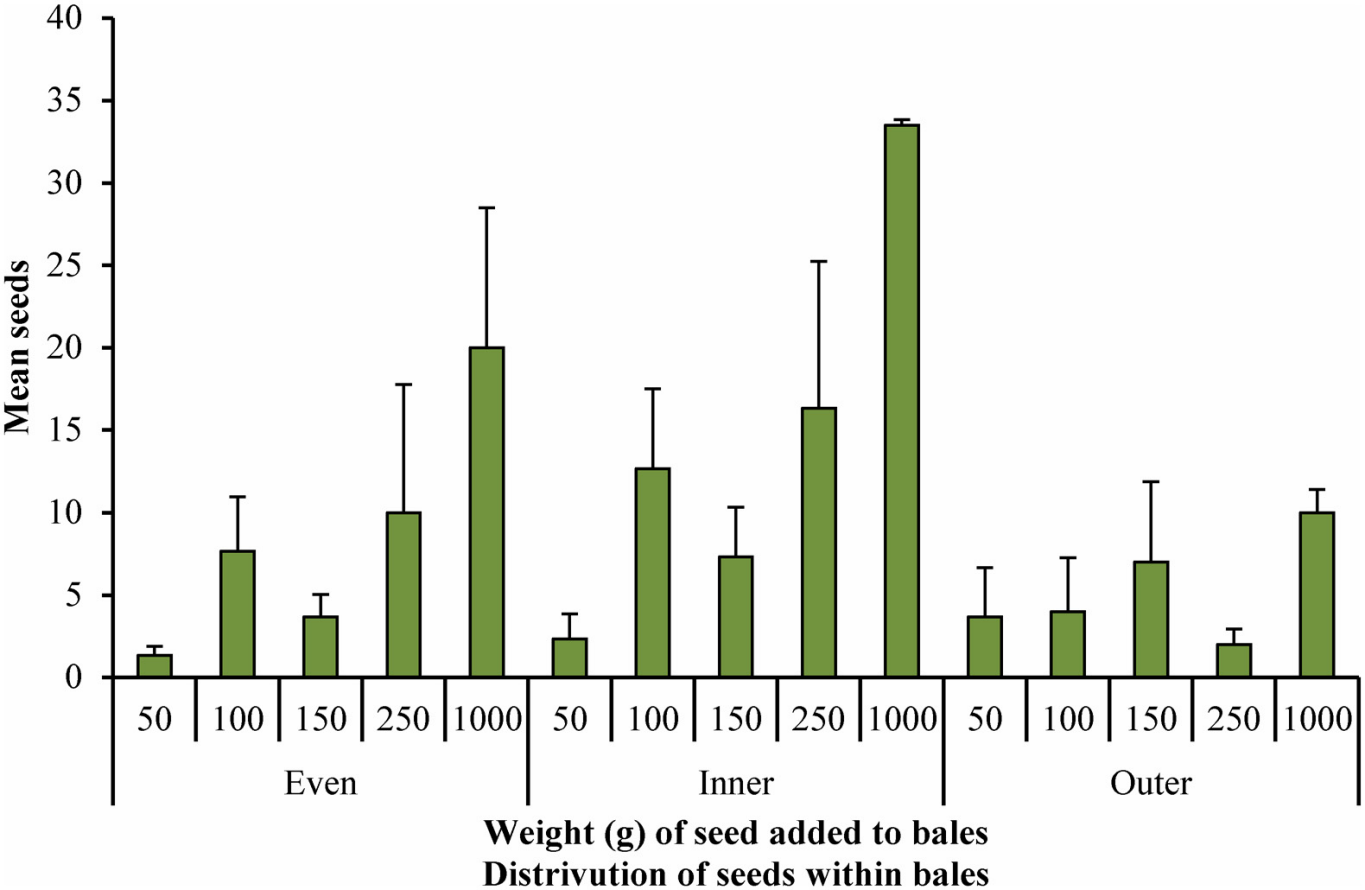
Numbers of seeds found per bale in bales to which varying weights of seed were added, in three different distribution patterns, 5 cores per bale.

The mean and variance for the numbers of seeds found in each sub-sample of cores was calculated. These are given in Fig 3 and Fig 4 (S1 Dataset) and results for a regression of this data are given in Tables 4 and 5 (S2 Dataset).

**Table 4:** Results of regression for numbers of seeds, seven cores per bale.

Regression Statistics	Even	Inner	Outer
*R* ^*2*^	0.73	0.85	0.76
Adjusted *R* ^*2*^	0.70	0.84	0.74
Observations	14	14	14
Intercept (*b*_*0*_)	3.49 ± 1.85	0.68 ± 1.91	-0.24 ± 1.15
Intercept (*b*_*1*_)	0.05± 0.005	0.08± 0.005	0.04± 0.003
Regression P-value	0.000	0.000	0.000
*b*_*0*_ Lower 95%	-4.59	-7.63	-5.25
*b*_*0*_ Upper 95%	11.57	8.99	4.76
*b*_*1*_ Lower 95%	0.03	0.06	0.02
*b*_*1*_ Upper 95%	0.07	0.10	0.05

**Table 5:** Results of regression for numbers of seeds, five cores per bale.

Regression Statistics	Even	Inner	Outer
*R* ^*2*^	0.29	0.49	0.10
Adjusted *R* ^*2*^	0.23	0.44	0.02
Observations	14	14	14
Intercept (*b*_*0*_)	3.15 ± 1.62	5.49 ± 1.75	3.42 ± 1.08
Intercept (*b*_*1*_)	0.02 ± 0.004	0.03 ± 0.004	0.006 ± 0.003
Regression P-value	0.05	0.01	0.28
*b*_*0*_ Lower 95%	-3.89	-2.13	-1.28
*b*_*0*_ Upper 95%	10.18	13.12	8.11
*b*_*1*_ Lower 95%	0.0001	0.0102	-0.0055
*b*_*1*_ Upper 95%	0.04	0.05	0.02

For seven cores per bale, the fit of each regression (*R* ^*2*^) indicates a better than 0.70 fit of the data for all distributions, indicating that most of the variation in the results can be accounted for by the regression results and less than half is due to random error (Table 4). Inner distribution gave the highest *R* ^*2*^ value and the Even distribution the lowest.

The seed detection cut-off figures, indicated by the intercept (b_0_), were 3.49 ± 1.85 seeds for Even distribution, 0.68 ± 1.91seeds for Inner distribution, and -0.24 ± 1.15 seeds for Outer distribution. While this last result may seem anomalous, the absolute range given by the standard error indicates that at least one seed may be detected per bale. This indicates that it is possible to detect a minimum number of seeds in these bales of between approximately 1 and 4 seeds (rounded up).

For five cores per bale, the fit of each regression (*R* ^*2*^) indicates a less than 0.50 fit of the data for all distributions, indicating that less than half of the variation in the results can be accounted for by the regression results and more than half is due to random error (Table 5). Inner distribution gave the highest *R* ^*2*^ value and Outer distribution the lowest

The seed detection cut-off figures, indicated by the intercept (*b*_*0*_), were 3.15 ± 1.62 seeds for Even distribution, 5.49 ± 1.75 seeds for Inner distribution, and 3.42 ± 1.08 seeds for Outer distribution. This indicates that it is possible to detect a minimum number of seeds in these bales of between approximately 4 and 6 seeds (rounded up).

Overall, the number of seeds detected was positively, but not perfectly, correlated with weight of seed added to bales. The *R* ^*2*^ (adjusted) values indicate that reliability of detection of seeds ranges from low to moderate rate for five cores per bale, to moderate to moderately high for either seven or fifteen cores per bale. In most cases, the variance for each distribution is greater than the mean, indicating that the data are over-dispersed. This can be explained by the distribution of seeds within the bales being clumped or contagious, rather than being perfectly evenly dispersed. The p-values indicate a significant difference between the weight of seed added (<0.05) for all distributions at fifteen and seven cores per bale, and for Even and Inner distributions at five cores per bale. For five cores per bale, Outer distribution the p-value is not significant. Generally then, this result indicates that core sampling is a valid approach, but needs further investigation to confirm these results.

### Challenges to ‘evenly’ dispersing seeds in bales during this experiment

The seed morphology of *N*. *neesiana* affected how the seeds reacted or ‘behaved’ after being collected from the field. These seeds possess a long awn, a common feature of many grasses in the tribe *Stipae* [33, 34]. This appendage appears straight, without any bends, as the seed is developing on the seed head, but as it dries out it tends to become bent or twisted. This may occur either before or after seed fall. If it is sufficiently wetted by rain or another source of water, the awn is subsequently able to straighten out. As it dries out, it once again twists up. This alternate straightening and twisting due to water or moisture exposure can move the seed over the surface of the ground until the tip of it becomes lodged in a suitable germination site, such as a small crack in the ground. This type of movement is termed ‘hydrochory’ and is a feature of species whose seeds have hygroscopic appendages [35, 36].

When the seeds were collected from the plants, they were still attached to the parent plant via the panicle stem and awns were straight, indicating these structures were hydrated. It was noticed that after being stored for a short time (one day or so), the awns of these seeds became twisted and entangled with each other. When separation of the seeds into small clumps or individual seeds was attempted, this was found to be rather challenging. This had consequences for subsequent experimentation and analysis.

Prior to experimentation, seeds were weighed out as three replicates for each of five different weight categories (50, 100, 150, 250, and 1000 g). The awns of the seeds were strongly entangled, giving the visual appearance of large clumps, each of which measured several centimetres in diameter. These large clumps were carefully pulled apart as was possible, to ensure the awns remained attached to the seeds, when being weighed out into each of the required portions for experimental dosing of the bales. However, it was difficult and extremely time consuming to separate sufficient numbers of individual seeds for the purpose of weighing out quantities of seeds, since even the lowest weight (50 g) consisted of more than 6,500 seeds. In the field, the seeds were separated as far as was possible, in the limited time allowed (4 hours or so) to place the seeds in the rolled out windrows of clean hay, into small clumps that consisted mostly of a few dozen or hundred seeds (roughly estimated). These small clumps were sprinkled in the windrows as ‘evenly’ as possible.

Additionally, to ‘evenly’ disperse seeds along the windrows, it was desirable that the clumps of seeds be as small as possible, but this was difficult to achieve in practice. There was only a limited time to add seeds to the windrows, this being less than one day. Once the bales of clean hay were rolled out, it was undesirable that they remain in that state for more than one or two days. Any rainfall would have prevented the re-rolling of the bales for several days. This experiment was conducted during early autumn, a time of year when rain can be expected on most days. The result was that the seeds were dispersed in larger clumps throughout the bales than was desirable. This is reflected in the results obtained for the numbers of seeds found.

The equipment that was used to obtain the core samples allowed for at least 15 cores per bale to be collected in less than one hour, generally as quickly as 20 to 30 minutes. The time taken for pulling apart and sieving a single bale is not given in Thomas et al. (1984) or Conn et al. (2010), but it is unlikely that it would have taken less than this time to obtain samples from each bale. The bales sizes examined were also not stated in these articles; however, the bales sampled in this study were a minimum of 250 kg. Manually pulling this sized bale apart and sieving to find seeds could be expected to take in excess of one hour. The effort of core sampling is of a reasonably physical nature and there is variability of density within and between bales. Whilst coring was found to be more difficult with very dense parts of the bales and tended to be quite time consuming, if compared to dismantling an entire bale, the time taken to obtain core samples from each bale is considerably less. This suggests, therefore, core sampling bales to test for the presence of weed seeds is an area of study that should be further investigated.

Examination of the core samples, to enumerate all seeds of *N*. *neesiana* seeds that were present, took between 10 and 20 minutes per core. While this may seem to be somewhat time consuming, in most cases the 1 mm mesh sieve was able to detect seeds in only a few seconds. The subsequent examination of the material post-sieving was required to find the remainder of the seeds. In a situation where the only requirement is a ‘yes or no’ answer to the presence of the seeds of this species, the detection of seeds in a purely qualitative manner can be achieved quickly.

### Fodder waste fractions from large round bales

Belyea et al. (1985) and Buskirk et al. (2003) conducted experiments to determine the quantity of wastage from feeding large round bales to cattle. The former authors investigated the effect of storage of feed prior to feeding on wastage. The latter investigated the effect of devices used to hold bales, while livestock fed on the bales contained therein, on fodder wastage and feeding behaviour. Percentage of wastage ranged from a minimum of approximately 4% to a maximum of approximately 25% [30, 31]. The results for the possible numbers of seeds that would remain on the ground, from bales dosed with the seeds of *N*. *neesiana* at five different weight amounts and evenly dispersed within the bales, if these bales were to be fed to livestock are given in Table 6.

**Table 6:** Total numbers of seed per square metre remaining for each weight of seed added to bales

Authors	Waste fraction of bale	Remaining seeds per m^2^				
		50 g	100 g	150 g	250 g	1000 g
Belyea *et al*. 1985	0.12	11	21	32	53	211
	0.25	22	44	66	110	439
	0.14	12	25	37	61	246
	0.13	11	23	34	57	228
Buskirk *et al*. 2002	0.06	5	11	16	26	105
	0.04	4	7	11	18	70
	0.11	10	19	29	48	193
	0.15	13	26	39	66	263

Assumptions made in this investigation are that:

- each bale originally weighed 300 kg;
- the weight of the remaining factions for each seed weight is calculated from the percentages of waste;
- the numbers of seeds per gram is calculated from these fractions; and
- the bales are rolled out in a paddock covering an area of 75 m^2^ (1.5 x 50 m) for feeding to livestock.

Research into the second question has shown that, for some species, as few as 10 viable seeds per square metre may be able to achieve this outcome [37, 38]. In investigating the biology of this weed, Gardener (2003) assessed the germination rates of *N*. *neesiana* panicle seeds with the lemma removed, compared to those which did not have the lemma removed. It was found that the latter had lower germination rates, being approximately 48.5%, compared to 82% for the former.

Assuming that panicle seeds with the lemma still attached are incorporated into these bales, and taking the total seed viability of 48.5%, recalculating the number of viable seeds per square metre, we find that a bale with 1000 g of seed per bale would pose a significant risk for the establishment of a new infestation, even if only 4% of the bale remain unconsumed, since at least 35 viable seeds per square metre would be present in this amount of fodder (Table 7). This exceeds the minimum number of seeds, 10 per m^2^, found by other researchers that will initiate infestations [37, 38]. For bales with 100 g, 150 g or 250 g of seed, this situation is likely to occur where 10% or more of the bale remains unconsumed. For bales with 50 g of seed the risk is low, except where a significant proportion of the bale (25% or more) remains unconsumed. It can be shown, therefore, that most of these bales would be of a high risk for seed dispersal, if the indicated rates of non-consumption can be assumed.

**Table 7:** Numbers of viable seed per meter squared remaining for each weight of seed added to bales.

Authors	Waste fraction of bale	Remaining seeds per m^2^, 48.5% viability				
		50 g	100 g	150 g	250 g	1000 g
Belyea *et al*. 1985	0.12	5	10	15	26	102
	0.25	11	21	32	53	213
	0.14	6	12	18	30	119
	0.13	6	11	17	28	111
Buskirk *et al*. 2002	0.06	3	6	8	13	52
	0.04	2	4	6	9	35
	0.11	5	10	15	24	94
	0.15	7	13	20	32	128

These calculations assume that seeds are evenly dispersed within the bales. If bales in which seeds are unevenly dispersed (i.e., towards the inner or outer regions), are used to calculate the numbers of seeds remaining, the amount of baled material containing seeds would cover a smaller area of pasture, approximately one third of the total (25 m^2^). However, the numbers of seeds within this area would be at a higher density than for evenly dispersed seeds. The risk of establishment of a new infestation would still be present in this situation and it is likely that seed densities would be high enough to establish a new weed infestation.

## Conclusions

The sample size of 15 cores per bale is likely to require confirmation as an appropriate sample size, since the variance was large for all treatments and much larger than that used for other purposes. Statistical analysis indicates that for this experiment, approximately half this number of cores still gave a fairly reliable indication of seed presence in the bales, even when seeds were unevenly distributed throughout the bales. It is possible that if more bales had been available for sampling, even fewer cores per bale would be suitable for detecting seeds. Since more bales could theoretically be sampled in the same time as a single bale with the previously investigated methods, this method is worthy of further investigation. In the light of the finding, as a theoretical exercise, that bales contaminated with as little as 1000 g of the seeds of this weed are highly likely to initiate new infestations even where a relatively small a proportion of the bale is unconsumed by livestock, this investigation warrants further attention.

## Supporting Information

S1 Dataset Data1 for Paper2. (XLSX)

S2 Dataset Data2 for Paper2. (XLSX)
